# Abdominal Pregnancy1Published in the *New York Medical Journal*.

**Published:** 1907-08

**Authors:** John Egerton Cannaday

**Affiliations:** Hansford, W. Va.; Surgeon in Charge, Sheltering Arms Hospital


### Abdominal Pregnancy.[1]

By JOHN EGERTON CANNADAY, M. D., Hansford, W. Va.
Surgeon in Charge, Sheltering Arms Hospital.

[*Author's Abstract.*]

AFTER discussing the history of the lesion so far as the surgical knowledge of it is concerned, the author states that only during the last fifty years has its treatment been worthy of consideration. Lawson Tait has been the pathfinder in this as in some other subjects. The statistics of frequency vary widely according to the counts of different observers. About 8 per cent. of all cases of extrauterine pregnancy are said to be abdominal in character.

The symptoms are divided into those common to all varieties, and those peculiar to individual varieties. Of the first class are the reflex symptoms which belong to all normal pregnancies. The nausea and vomiting are commonly severe and begin usually early in pregnancy. Two symptoms specifically point to extrauterine gestation. They are the bloody discharge, and the ab-

1. Published in the *New York Medical Journal*.

dominal pains which are as a rule colicky and sharp; they start from the region of the tumor and radiate downward and outward These pains may begin about the first of the second month and last throughout pregnancy. The acme of their severity is about each menstrual period, and there may be an intermission of enti[illegible] freedom from them between the periods. During these attacks of pain the abdomen may be swollen and tender to the touch. The pulse is accelerated, but there is no temperature rise. The bloody discharge from the uterus occurs in a majority of patients. This phenomenon is usually accompanied by pain and the expulsion of the decidual membrane, the discharge being due to rupture of the decidua, of a seropurulent, coffee colored or reddish nature, and may be apparently so profuse as to call for tamponade. In the primary abdominal type there may be no disturbance of the menstrual function. The return of the menses is indicative of fetal death. The rectum may be irritable, and pulsation can often be elicited by vaginal palpation. The most typical symptom is metrorrhagia coincident with the symptoms of pregnancy in its early stages. If associated with this is a discharge of decidual tissue one should expect extrauterine gestation.

False labor may be premature, happening at the seventh or eighth month, but usually makes its appearance at term, rarely afterward. At the same time the patient has intermittent pains analogous to true labor pains. The cervix does not become obliterated but dilates sufficiently for the entrance of one or two fingers. After the decidua is expelled the pain ceases and does not return unless there has been a rupture of the fetal sac. The signs of labor will disappear, and milk will come in the breasts.

The symptoms of rupture are sudden and severe pain radiating over the abdomen, rapid weak pulse, air hunger, shock, and other concomitants of hemorrhage. There is apt to be nausea, hiccough, and extreme tenderness of the abdominal walls. The escape of the fetus from the tube without much loss of blood is marked by severe pain referable usually to the side, tenderness of the abdomen, and often a temperature rise. The rupture may be spontaneous or provoked by some slight trauma.

*Physical Diagnosis.*—The os and cervix are often soft, and either firmly confined by adhesions or pushed entirely out of their natural position by the rapidly enlarging cyst. Fetal pulsation may be felt through the vaginal wall, and the fetus can at times be outlined in the same way. There are two tumors, one of which is usually situated to the right or left of the median line. A sulcus between the adventitious body and the cervix can be made out. In some cases the fetus is palpable through the abdominal wall. On manual examination of a cyst containing a dead fetus of considerable size crepitation of the bones may be obtained. The uterus remains stationary in size after the fourth month. Fetal heart sounds and movements are discernible after the fifth month

*Diagnosis.*—The diagnosis is nearly always difficult, and cannot be made with certainty during the first period. At that time diagnosis of probability constitutes an ample reason for surgical intervention. It may be taken for ovarian cysts, fibroid tumors, several forms of salpingitis, and hematocele. It may possibly be differentiated from these by the history, the malposition of the uterus, and the disturbances of pregnancy. In the second period of pregnancy diagnosis is not so difficult, but it is nearly always impossible to distinguish one variety from another. In making a diagnosis we have what we can elicit from the story of the patient in her own words, her replies to minute questionings, and a physical examination. After the escape of the fetus from the tube and the beginning of the secondary abdominal type, the acute symptoms may subside, but there are apt to be recurrent attacks of pain. An apparently normal condition necessarily tends to throw the physician and patient off their guard. The diagnosis is naturally difficult, because of the irregularity of the symptoms, the frequency with which it is simulated by other conditions, and the ease with which the bleeding with or without expulsion of the decidua may be taken for an ordinary abortion. Probably there are few conditions more plain to the careful observer than a typical case of exfetation, but comparatively few cases are typical.

The diagnosis of abdominal pregnancy is rather rarely made prior to false labor, for the reason that the physician's attention is seldom called to the case. We should regard sudden collapse associated with pallor and other symptoms of intraabdominal hemorrhage in any woman having a possibility of pregnancy as prima facie evidence of a ruptured ectopic gestation sac. A period of amenorrhea usually precedes the bloody discharge which does not correspond in nature nor necessarily in point of time with the natural monthly bleeding. Important points relative to the bleeding are the color, the persistence, and the presence of membrane or pieces of membrane. Among the most characteristic symptoms are the variable period of amenorrhea, irregular uterine hemorrhage, pelvic pain, and discomfort, and the shedding of the uterine decidua. This pregnancy is like a mine, ready to explode with a moment's notice, and it is highly important that the patient be in easy reach of competent surgical skill at all times. It is nearly always best to appreach these pregnancies by a median laparotomy. Complete removal of fetus, membranes, and placenta is highly desirable. By reason of dense adhesions great danger of hemorrhage or dangerous condition of the patient this procedure will at times be impossible. Under such circumstances the edges of the opening in the sac shouid be sutured to the parietal peritoneum and the sac carefully drained. The placenta in such cases will come away gradually by fragments, and in two or three weeks its exfoliation will have been complete. Surgical intervention should take place as early as possible after the death of the fetus. If the cyst in such case is in the cul-de-sac vaginal section is appropriate; after the extraction of fetus and placenta the cavity had best be packed with a five percent. iodoform gause. I wish to urgently emphasise the absolute necessity for removal as early as a diagnosis can be made, and the stringent indication for immediate operation when we see a pregnant woman showing symptoms of intraabdominal hemorrhage.

The author reports a case in which the diagnosis was made prior to rupture, but operation was not resorted to until a short time after rupture had taken place. Although the placental implantation was very extensive a complete operation was performed. Although the patient was in a very bad condition from hemorrhage and shock, under prompt stimulation she reacted and made an excellent recovery, the wound uniting by primary intention without drainage.

The writer summarises his paper as follows:—The greater frequency of ectopic pregnancy than the number of observed cases would lead us to believe: the usual irrelevant and atypical nature of the symptoms; the difficulties in the way of making a diagnosis and necessity for a careful study of the cases, in which the condition might be suspected. both in its present and past aspects; the importance of studying the character of the uterine discharges; the association of this with pelvic pain and discomfort, and the signs of pregnancy; the advantages of prompt operation, removal of blood and other debris by dry sponging without irrigation, thorough hemostasis, and the closure of the wound without drainage.

